# Notes on the Treatment of Flesh Wounds in Time of War in the Surgical Formations of the Army

**Published:** 1918-03

**Authors:** Pierre Duval

**Affiliations:** Consulting Surgeon of the 7th Army


### NOTES ON THE TREATMENT OF FLESH WOUNDS IN TIME OF WAR IN THE SURGICAL FORMATIONS OF THE ARMY

PIERRE DUVAL
Consulting Surgeon of the 7th Army.

The treatment of flesh wounds in time of war is determined by the bacteriological conditions under which they develop.

1. All war wounds must be considered as infected;

2. In the initial period the wound is only superficially contaminated by the organisms : according to bacteriological and histological examination this period lasts only from 6 to 8 hours : practically, it may be said to average 12 hours, from the point of view of the surgical treatment of the wound.

In a second period, after the 12th hour, the surface contamination changes to a deep infection of the wound.

The dominant factor in the multiplication and deep dissemination of the organisms is the presence of tissues devitalized by the traumatism.

It has been demonstrated that an early intervention consisting in the removal of the foreign bodies from the mortified and contaminated tissues converts the primarily infected wound into a practically aseptic surgical wound, capable of immediate union.

When the excision of the mortified tissues has not been or could not be complete, the wound must be regarded as infected and as demanding a progressive sterilization. When a wound can be operated only after a delay of at the least 15 hours, it must be considered not merely as contaminated but also as infected.

1/*a* — In 100 flesh wounds operated after a delay of at least 15 hours, 85 to 90 o/o can be operated under conditions which will allow of primary suture. Complete success is obtained in from 90 to 95 o/o of these cases. The failures are generally partial and not serious, the total removal of the suture is rarely necessary.

1/*b* — The delayed primary suture is applicable to those wounds which have been operated in the advanced military formations, by the removal of foreign bodies and the excision of mortified tissue, but which have not been sutured. During a recent offensive, out of 100 cases thus operated and evacuated to a base hospital, 80 o/o were sutured, the average delay of the suturing being 11 days. The delayed primary suture resulted in from 4 to 5 o/o of failures.

1/*c* — Secondary suture after a progressive sterilization of the wound was performed in about 15 o/o of the cases. This resulted in from 4 to 5 o/o of partial failures.

1/*d* — All the wounded, upon whom primary suture was performed, were retained for at least 15 days in the surgical ambulance where they were operated. This retention under the care of the surgeon who has performed the suture is now considered indispensable.

If it is impossible to keep the wounded man in the formation, delayed primary suture must be preferred. In a period of calm it is possible to keep 85 o/o of the wounded.

1/*e* — In a period of calm 20 o/o of the wounded may be evacuated.

During a great rush of wounded, the number of operated cases which must be evacuated varies with the total number of the wounded, and the capacity of the hospital.

In the last 2 offensives we have been able to perform primary sutures on 30 o/o of our wounded, and to retain 30 o/o : thus we have been obliged to evacuate 60 o/o of our cases and to perform delayed primary suture in the base hospitals.

1/*f* — After the two first engagements in the Battle of Flanders, 700 wounded were evacuated to Amiens to undergo delayed primary suture.

The numbers given by the Surgical Center at Amiens were as follows :

32 o/o were returned to their Corps after 7 days' leave and a treatment of 3 weeks.

30 o/o were returned to the dépôt after a month's treatment and a month's convalescence.

28 o/o were considered as recuperable after more than 2 months. *Total* of 90 o/o recuperated — of which 62 o/o, with a maximum delay of 2 months.

*Indications for primary suture.*

1. The possibility of operating within from 12 to 15 hours after the wound is received.

2. The possibility of removing all foreign bodies (splinters, bits of clothing) and of excising all the devitalized tissues.

Primary suture can be performed by an operator who is experienced in this branch of surgery far beyond the delay of 12 or 15 hours. For those who are only beginners in war surgery, I think a delay of 12 hours should not be exceeded.

In a period of calm primary suture can and ought to be performed on nearly all who are suffering from flesh wounds : this is the preferable method; it is the method that gives the quickest and the best results.

The retention of the patient in the formation where he has been operated and under the unremitting care of the surgeon who has taken the responsibility of the primary suture, is imperative for a minimum period of 15 days.

Primary suture is not practicable for all the wounded during the time of a great rush. Delayed primary suture must often be substituted. And the wound closed on the 3rd, 4th, or 5th day or later without intermediate chemical sterilization.

The shortage of hospital accommodation at the front is an indication for delayed primary suture.

In a period of calm delay is quite unnecessary : during great activity, it gives a result nearly equivalent to that of primary suture. The incomparable benefit of primary suture is thus extended to all the wounded. The delayed primary suture permits of the most complete treatment for all who require it — that is to say, primary union.

During the offensive in Flanders in 30 o/o of the cases primary sutures were performed as against 90 o/o in a period of calm. 60 o/o of flesh wounds were treated by delayed primary suture. This disparity between the two figures varies of course with the number of wounded and the possible amount of hospital accommodation.

The indication for delayed primary suture is the bacteriological examination of the wound. Such an examination during great activity can only be quantitative and not qualitative.

The examination by smears with the Carrel technique is the method employed during periods of heavy surgical work.

It is always necessary, however, to make at the time of this quantitative examination, a few culture tests as to the nature of the organism, which is a much more important consideration than the quantity.

These cultural examinations will show in a general fashion the nature of the germs which may be found in a series of wounds received on the same field of battle.

When the wounds present a very satisfactory clinical aspect (bright red and uniform coloration of the muscles), absence of pockets, and perfect elasticity of all the tissues, the examination by smears may be considered sufficient. Should the clinical aspect of the wound not be absolutely favorable, especially if the wound is ragged with numerous sinuses, a cultural examination is indispensable.

By employing Carrel's numeric method, every wound can be sutured that presents 0 to 1 microbe in 5 fields. From this point of view, I have personally, in an advanced surgical centre, made the examination of 61 wounds, which had arrived after a delay of 24 hours. 47 were aseptic, 34 showed 0 microbe, 12 showed 1/10 to 2 microbes (bacilli, diplococci).

All these shell wounds since operation had been covered merely with sterilized gauze.

It was therefore possible to suture about 80 o/o of these wounds. In 80 o/o of cases, after surgical treatment (removal of foreign bodies, excision of devitalized tissues) the wound may be sutured. The simple aseptic dressing assures a practically aseptic condition of the wound during several days and allows of retarded primary suture.

*Location of wounds; field of battle.*

To this general recommandation certain observations must be added.

In some cases the location of the wound necessitates a very cautious use of primary suture.

Thus wounds of the deep muscles of the buttock are particularly liable to gas gangrene, as, to a less degree, are those of the anterior region of the thigh and the calf of the leg. A surgeon who has had little experience, should in such cases have recourse to delayed primary suture.

On the contrary, in scalp and face wounds, the limits of the primary suture may be much extended.

Before deciding in favor of primary suture, the surgeon must take into account the character of the field of battle.

There are on the western Front regions were the bacterial flora is peculiarly virulent, and experience proves that there are sectors of the Front where failures follow primary sutures more frequently than in others.

When the wounded are brought in from a reconquered area extreme caution must be observed and delayed primary suture during the first days must be preferred, until bacteriological examination can determine the gravity of the contamination.

*Indications for secondary suture.*

Secondary suture is indicated for all wounds that have been infected. Three types of cases are to be considered :

1. Patients who have come into the surgeon's care after from 15 to 20 hours have elapsed.

2. Patients who have arrived in less than 15 hours, but because of the nature of the wound it has been impossible to make the necessary excision or to extract all the foreign bodies.

3. Patients whose wounds have been primarily sutured, but the results have been total or partial failures.

In these three instances the wounds must be treated by progressive sterilization and secondary suture.

The moment for suturing the wound is indicated by repeated bacteriological examinations.

Today it appears undeniable that for secondary suture Carrel's count method is insufficient. The examination by culture is absolutely necessary for all wounds.

If the count examination suffices and is adopted as the ordinary method for the delayed primary suture in times of great activity, the cultural examination added to the count examination is necessary for the secondary suture which may be purposely delayed for several days.

For streptococcic wounds this principle is of first importance. At present, a wound infected by streptococci can be sutured only after being entirely freed from these organisms[1].

*To resume*, — in order to perform secondary suture, the bacteriological examination must be made in the following manner:

*a*) Examination by culture on the arrival of the wounded in order to determine the nature of the germs.

*b*) Count examination by Carrel's method during the desinfection of the wound.

*c*) Cultural examination at the moment when the wound appears numerically free from microbes in order to be certain of its aseptic condition.

The suture can then be performed with security.

### III. — Operative Technique of the Primary Suture.

The important step in primary suture is the excision of the devitalized tissue. After an extended incision, two processes follow: the surgical cleansing of the wound and the excision *en bloc* of the devitalized tissue, as in the surgical excision of a tumor.

The excision with curved scissors or with the knife, is performed on the surface of the wound, in the direction of the healthy tissue.

This technique is open to criticism.

The infected instrument certainly infects layer after layer of the healthy tissue.

The excision *en bloc* is undoubtedly the best process, but its difficulties and drawbacks must be considered. There is a tendency to exceed the limits of the area to be excised and this has caused important loss of substance in the muscular fiber.

Only after long experience can this technique be followed with security.

To avoid possible vascular or nervous section a very precise anatomical knowledge is necessary.

It is relatively easy of application in surface and penetrating wounds; in the deep setons it is difficult to excise all the passage by the two incisions made at the orifices.

1. With a view to early secondary suture it may be well to employ the autovaccine method of Wright in the treatment of streptococcic wounds.

The excision must be continued until healthy tissue, which bleeds normally, is reached.

The functional restoration of the tissues, which may sometimes be obtained by primary suture, may justify the section of certain muscles in order to facilitate the excision; but the possibility of failure must always be taken into account and the uncertainty of the results must always limit the zeal to operate.

The method of Legrand, wich consists in pouring into the wound a solution which colors the devitalized tissue and marks the destruction of the healthy tissue, may assist the excision. I have had no personal experience with this method.

When the excision is made, the tissues are washed and rubbed with ether, then the suture is made layer by layer. Muscles and aponeuroses are sutured with catgut : the skin, by any method.

Hemostasis must be effected with great care. Drainage by rubber or by a strip of gauze should be attempted only when a bacteriological control is necessary after the suture. A capillary drain can be slipped in between two of the stitches. At the end of six hours a test is made by means of a pipette. At the end of 12 hours, the culture will show results and if necessary a larger drain can be established.

The early total disunion of the wound should be exceptional. But the fact always remains that the excision of war wounds followed by immediate suture is very difficult surgical work, difficult in its extent and difficult in its execution. The immediate results of operation demand the closest surgical watchfulness.

Such surgery can be practiced only by professional surgeons who have long experience in war work.

In certain cases of very extended injuries, a primary suture of a part of the wound may be practiced.

The main portion of the wound is sutured; one portion is left gaping, with or without a drain; if the bacteriological examination permits, this portion is treated little by little by delayed primary suture and, if it is found necessary, by a progressive chemical sterilization and a secondary suture.

This is a precautionary method which gives excellent results.

### The Operative Technique of the Delayed Primary Suture.

The first act of the operation — removal of foreign bodies and excision of the wound — is exactly the same as in primary suture. The wound, cleaned with ether, is left open. The surface is covered with aseptic gauze. The suture is performed as soon as possible. The earliest moment is the best. But the delayed primary suture can be performed up to the 8th day.

The delayed suture, if performed on normal tissue without granulations, requires no fresh excision.

The wound is vigorously rubbed with ether. The different layers of tissue are separated, one from the other, and the süture is made layer by layer.

Delayed primary suture is merely the second stage in the primary suture — only retarded.

In a doubtful case a capillary drain can also be inserted in the passage, to allow of bacteriological tests.

### Operative Technique of the Secondary Suture.

Secondary suture is performed on wounds where the tissues have already granulated, that is, on wounds with diseased tissue.

2 techniques present themselves : 1. Suture of the skin over fleshy granulations. 2. The excision of the layer of fleshy granulations and of the sclerosed tissue surrounding them.

The suture of the skin over the fleshy granulations is effected by the simple excision of the cutaneous edges. The skin is then detached to the necessary extent and the edges are stitched together The skin must not be devitalized by this operation.

The excision of the granulations is effected by the removal of the whole layer with the knife and the excision of the underlying layer of tissue. Normal tissues which can be sutured layer by layer are thus layed bare.

It is a mistake to currette the granulations, for sclerotic tissues are left and numerous vessels are opened producing an hematoma under the suture.

Personally I never perform a secondary suture over pathological granulations. Such a suture contains sclerotic tissue, the presence of which gives an inferior functional result. I infinitely prefer a secondary suture after a surface excision ; the functional result obtained seems to be much better.

### Length of Treatment and Disability According to the Different Methods of Suture

Primary suture requires an average hospitalization of 3 weeks : 2 weeks in the front hospital where the operation has been performed, and 1 week in a base hospital.

The delayed primary suture prolongs the treatment by the number of days which elapse between the first intervention at the front and the suture at the base : 10 days more must be allowed, making in all a total of one month.

Secondary suture demands a much longer hospitalization and usually requires a course of physiotherapy at some resort. Whatever method may be adopted for the progressive disinfection of a wound, an average term of 3 weeks is required.

In our army streptococcic infection of wounds is responsible for the majority of cases that must be treated by this method. It is only at the end of 18 to 22 days of sterilization that the suture can be attempted — 15 days at least are required for the complete healing of a secondary suture.

The physiotherapic treatment necessitated by the muscular sclerosis inevitably produced during the whole septic period, lasts 2 weeks or more. The length of convalescent leave varies also with each mode of suture.

*Primary suture.* — 7 days leave and return to the Corps in more than half the cases.

*Delayed primary suture.* — 7 days leave and return to the Regiment in 32 o/o of the cases. 1 month's convalescence in 30 o/o of the cases.

*Secondary suture.* — On an average 2 to 3 months' leave.

Thus it is seen that the primary suture of a flesh wound renders a man recuperable and permits of his direct return to the regiment in nearly 66 o/o of the cases after from 4 to 5 weeks of disability. The delayed primary suture gives the same result in 32 o/o of the cases. In 30 o/o of the cases the wounded man can return at the end of 2 months to the dépôt, but not to the regiment. A total of 62 o/o recuperate within 2 months.

In the last two offensives (Flanders, Malmaison) by the use of these two methods — primary suture and delayed primary suture — at the end of 5 weeks 85 o/o of flesh wounds were cured; the men required only a short convalescence before return to service.

Secondary suture renders a man fit only after a minimum period of 3 months.

It must be remembered, in considering this result, that at present in the French Armies, secondary suture is reserved for only those cases which are most serious by reason of the nature of their infection or on account of the disturbances caused by the wound.

The quality of healing obtained varies accordingly as it results from primary or from secondary suture.

Immediate primary or delayed primary suture procures functional restoration to the maximum extent compatible with the operative resection of the tissues.

In the large majority of cases, the patient suffers no diminution in his working or fighting capacity.

Secondary suture on the contrary, owing to the muscular sclerosis which is produced during the infected period, gives infinitely inferior results. The majority of the wounded treated by this method suffer a diminution of working and fighting power.

### IV. — Chemical Sterilization of Recent Wounds.

It is only a short time since all war wounds and especially all flesh wounds were considered to be inevitably infected after operation. Chemical sterilization was consequently thought to be necessary in all cases.

Today it is employed only in infected wounds, which it has not been possible to operate suitably, or in those which could be operated only during their infected period. Thus the chemical sterilization of wounds is not an invariable necessity : from henceforth it may be looked on as a last resort to which recourse must be had under regrettable circumstances.

A question of highest importance is settled — the uselessness of the so-called retarding cheminal process. (In France, *Poudre de Vincent*, *Baume de Mencière*, etc.) Before operation, such preparations are not only useless, but sometimes harmful, according to the method of their application. (*Poudre de Vincent* is introduced by insufflation.) They are useless after operation because the aseptic dressing insures sufficient asepsis.

As to the chemical treatment of recent flesh wounds, it would be impossible for me to review all the suggested methods. I will give my own impressions. For 3 months we have studied comparatively the following methods from the point of view of their bacteriological effect and of their healing powers : Carrel-Dakin solution, Wright's hypertonic solution, chloride of magnesium, and the sun's rays.

When the wound was well opened, containing nothing that could keep up suppuration — foreign bodies, bits of clothing, splinters, etc. — and when there were no recesses to harbor stagnant pus we obtained the same results from nearly all methods.

Nevertheless, there are, we believe, certain special indications for the use of these different chemical substances.

In wounds with gangrenous surfaces the best results are undoubtedly obtained with the hypertonic solution of Sir Almoth Wright.

By the digestive process thus induced, the gangrenous surface is changed in the space of from 36 to 48 hours to a rosy surface freed from all devitalized matter. The effect produced is remarkable. But it has seemed to us that the prolonged use of the hypertonic solution gives results inferior to those produced by other cheminal agents. Carrel-Dakin, ether, or the sun's rays applied from the moment that the wound has been cleansed by the hypertonic solution, give a more rapid and a more complete sterilization than does Wright's solution. It has seemed to us that a wound treated only with hypertonic solution, although rapidly brought to a comparatively aseptic condition, did not progress quickly beyond that point but required long and difficult treatment to make it ready for suture.

In a wound with a gangrenous surface, the Carrel-Dakin or the ether treatment cleanses at first less quickly than does Wright's solution. The best formula in these cases has seemed to us to be : Wright for 48 hours, then Carrel-Dakin.

The solution of chloride of magnesium has in itself the immense advantage of being cyto-phylactic and its definite action is slower than that of Carrel. The objection to ether is that it is a rather scarce product. The sun is not always at the surgeon's disposal.

All of the different liquids have been used with the same dressing technique. Carrel tubes were placed in large numbers in the wound and the solution injected into the tubes every 2 hours.

For recent wounds, this comparative study has led us to the following practical conclusions.

I. The flesh wound must contain nothing which can keep up the suppuration (foreign bodies, splinters, or closed pockets).

When the bacteriological examination shows an increase or an even persistance of the infection, the existence of one of these causes is proved beyond doubt. In order to put the wound in a favorable condition, an operative revision of the wound is necessary.

II. The best method for the employment of these liquids is that of Carrel — numerous Carrel tubes and intermittent irrigation of the wound.

III. The frequency of the dressings is of supreme importance, as is also the cleansing of the skin of the limb. Lathering with oleate of soda is of prime importance, for a septic condition of the skin may keep up the infection of the wound.

IV. When the wound is in a favorable condition, when the dressings are frequently, carefully, and cleanly made, at but slightly differing intervals, any of these chemical treatments puts the wound into a condition capable of undergoing the secondary suture.

V. I have unfortunately no data, based upon a prolonged examination of the wounded, that would permit me to judge which of these different treatments gives the best quality of functional recovery.

Those patients who have undergone primary suture should never be evacuated immediately.

The wounded who have been prepared for delayed primary suture — that is to say, those who have undergone the first part of the complete operation (removal of foreign bodies, excision of mortified tissue, surface aseptic dressing, without suture) — can and ought to be evacuated to the surgical center as soon as possible (6 or 8 hours after the operation).

The length of the journey to the suturing center may be from 6 to 10 hours (in Flanders it took 8 hours; in the Aisne, 6 to 7 hours).

The length of the journey could without danger be greater. In the case of a rush of wounded, greater than the surgical capacities of the Army, non-operated flesh wounds may be immediately evacuated in special very rapid trains to the base centers. The wounded man should come into the surgeon's hands in the course of 15 to 18 hours after he has been wounded.

In the last two offensives the Army Medical Service had organized special very rapid trains having priority of passage to take these wounded to the specially prepared centers which were equipped by large numbers of surgeons capable of operating within a few hours on the wounded arriving from the front.

These centers were never employed, because the Army was able to operate all the wounded.

### VI. — The Present Organization of Surgical Services in the French Armies.

The following facts concerning flesh wounds must be borne in mind.

*a*) They are the most numerous.

*b*) When well operated, they heal in perfect condition.

*c*) Of those suffering from flesh wounds 80 to 90 o/o are recuperable ; 62 o/o of them within 2 months.

It follows, therefore, that flesh wound surgery is perhaps the most important.

At periods of a great rush of wounded this surgery merits the exclusive attention of a special organization.

*Clearing Centers.* — A man suffering from a flesh wound should be considered as a special case and immediately after passing through the clearing station, should be furnished with a special label and sent to a special operating center.

*Operating Centers.* — Operating Centers for flesh wounds are divided into three series.

*a*) An advanced series for the most urgent cases — surgery for the removal of foreign bodies, for excision without suturing or bacteriological examination ; in this series only a proportion of the wounded can be operated. The majority will be directed at once from the clearing station to the second line centers.

*b*) Second Line Series. A considerable operating center. This may be either an isolated center, or it may form a special part of a large front hospital.

A surgical service for flesh wounds must include a large number of surgical units, of operating tables, a radiographic outfits, and a bacteriological laboratory. For an Army Corps in action, 15 surgical units, 10 operating tables, 4 radiographs, and a laboratory must be allowed.

In periods of calm this formation performs primary sutures. In times of engagement, it practices surgical excision without suture.

*c*) Third Line Series for primary retarded suture. These require : a very large receiving capacity, a very numerous hospital staff, and a laboratory very fully mounted in staff and material.

These three series are directly related to each other. When the number of men suffering from flesh wounds surpasses the operative capacity of the Army, when it has been impossible to operate all these wounded in the 1st, and 2nd line series, they will be at once directed to the 3rd series or to a 4th for operation.

In these circumstances, special and very rapid trains with the right to priority of passage should be organized in order that the wounded may be operated with a maximum delay of 18 hours after the wound is received.

